# The development of pressure across membranes in Donnan systems

**DOI:** 10.1038/srep14695

**Published:** 2015-10-12

**Authors:** Asher Ilani

**Affiliations:** 1 Department of Medical Neurobiology, The Institute for Medical Research – Israel-Canada, The Hebrew University-Hadassah Medical School, P.O. Box 12272, Jerusalem 91120, Israel

## Abstract

The pressure that develops between the two sides of a Donnan system is equal to the difference between the osmotic values of the two solutions, even though permeant ions may constitute a significant part of that difference. This is amply documented for the case of membranes that allow water movement through them by single molecules diffusing in isolation or in series through specific proteins (such as aquaporins). In this article, the development of pressure was analysed for a system in which membranes contain a few bulk aqueous pores that prevent charged polymers from entering them due to their size. It is shown analytically that the pressure that develops by the action of the electric field on the net charges in the pores is equal to the difference in the osmotic values of the solutions contributed by the permeant ions. Thus, the sum of the pressures that develop in the system due to the action of the electric field in the pores (a pushing force) and the concentration of the impermeant polymers at the interface (a sucking force), accounts for the total colloid osmotic pressure in these systems.

The Donnan system was first described by F.G. Donnan over a century ago^1^ and has since been included in classical textbooks on general physiology^2^. A Donnan system at equilibrium is characterised by two phenomena: 1) the presence of an electric potential difference between the two sides of the membrane, which establishes equilibrium for the permeant ions across the membrane, and 2) the appearance of a pressure difference between the two sides of the membrane that establishes equilibrium for water. A diagram of a Donnan system at equilibrium is shown in Fig. 1. The potential difference across the membrane is equal to the equilibrium potential for the permeant ions as defined by the Nernst equation:


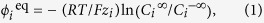


where *ϕ*_i_^eq^ is the equilibrium potential for permeant ion *i*, *C*_*i*_^*–∞*^ and *C*_*i*_^*∞*^ are the molar concentrations of permeant ion *i* far from the membrane in solution 1 and solution 2, respectively, *R* is the gas constant, *T* is the absolute temperature, *F* is the Faraday number and *z*_*i*_ is the valence of ion *i.* Here, ideal conditions are assumed in that the activity coefficients for all the solutes are taken to be unity. The solution on side 2 also contains a charged polymer of valence *z*_*P*_ at a concentration *C*_*P*_. The difference between the osmolarities of the two solutions depends on the concentrations of all the solutes (equation 2):


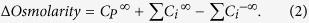


The course of the potential across the membrane is well understood when the membrane is a classical bimolecular lipid membrane (BLM) that is inherently permeable to water^3^ or whose permeability to water can be increased significantly by specific water channels, referred to as aquaporins^4^. A BLM may contain channels or carriers that render it permeable to various ions but impermeable to certain charged molecules. In this case, the membrane can be considered a capacitor and equilibrium is achieved when the charge in the capacitor corresponds to the equilibrium potential for the permeant ions.

When the membrane consists of a porous partition, however, the progress of charge separation and the course of the potential drop across the membrane have not been analyzed in the context of a Donnan system. In this article, the analysis is limited to cases where the membrane contains relatively few pores, so that most of the potential difference occurs within the pores. This implies that the concentration of the impermeant charged polymer at the interface is not significantly higher than its concentration farther away. For the system to attain equilibrium for water, a difference in pressure must be present between the two sides of the membrane that is exactly equal to the difference between the osmotic values of the two solutions—the colloid osmotic pressure. Otherwise, it could give rise to a *perpetuum mobile*. Nevertheless, the physical forces that generate this pressure remain unclear when the actual concentration of the impermeant polymer at the interface of the pore with solution 2 is only a fraction of the difference in *osmolarities* between the two solutions. The surprising resolution of this problem is presented below.

The Poisson electric equation was solved numerically for the bulk water-filled pores within the membrane. The system is homogenous with respect to the *y* and *z* coordinates, and so the Poisson equation takes the following form:


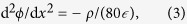


where *ϕ* is the electric potential, *ρ* is the charge density, 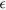 is the permittivity of free space and 80 is the dielectric constant of water. The details of the numerical analysis for a particular Donnan system are presented in the Supplement.

The numerical analysis of several Donnan systems showed that in the concentration range of a fraction of a millimolar and above, the total change in *ϕ*, *ρ and P* occurs within the pore, less than a few hundred Å from the interface (see Fig. 2 and Supplementary Figure S1).

The first surprise to emerge from the numerical analysis was that the action of the electric field on the charge in the water within the pores generates a pressure that is equal to the difference between the osmotic values of the two solutions contributed by the permeant ions.

The second surprise was that the above can be established analytically using the Nernst equation, in symbols (see the second part of the Supplement.).


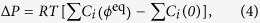


where Δ*P* is the change in pressure in the pore from the point where *ϕ* = 0 (at x < −1000 Å), to where *ϕ* = *ϕ*^eq^ (at *x = 0*). This expression is similar to the familiar van’t Hoff’s equation, and states simply that the pressure which develops in the pore toward its interface with solution 2 is equal to the contribution of the permeant ions to the difference between the osmotic values of the two solutions. In fact, it is equivalent to the *contact value theorem* first derived by Israelachvili (p. 300)^5^.

Thus, the pressure that develops across a porous membrane in a Donnan system has two components: a pushing force from side 1 to side 2 driven by the action of the potential gradient on the charges in the pores, and a sucking force acting from side 2 that originates from the concentration of the impermeant polymer at the interface of solution 2 with the pores^6^.

Equation 4 can be generalised to apply to any point along the pore (up to the value of *ϕ* at x = 0),


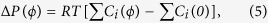


implying that all along the pore, water is in equilibrium with the solution on side 1.

The appearance of forces between charged surfaces separated by solutions is dealt with in detail by Israelachvili^5^.

## Discussion

The pressure that develops in a Donnan system is equal to the difference between the osmotic values of the two solutions and is unaffected by the degree of permeability of the membrane to the permeant ions even though these may contribute substantially to the difference between the osmotic values of the two solutions. Thus, a Donnan system seemingly challenges the concept that for a membrane to be able to develop a pressure that matches the full difference in osmotic values between two solutions, it must be an ideal semipermeable membrane. Instead, for a membrane to develop the full difference between the osmotic values of the two solutions, it is necessary only that there is no *net* solute flow across it. This is the case when the membrane is ideally semipermeable and there is no flow of solutes due to impermeability, or in a Donnan system where no *net* flow of solute occurs due to the absence of a driving force. When the equilibrium is disturbed in a Donnan system, for instance, by the addition of a salt composed of permeant ions to the side containing the impermeant charged polymer, the pressure across the porous membrane can drop even though the difference between the osmotic values of the two solutions increases^7^.

Unlike the BLM membrane, the case of water-filled pores has been considered mysterious. The analysis presented here partially resolves this enigma when the pore-area cross section is a minute fraction of the total membrane area.

What remains unsolved is the mechanism behind the pressure that develops in Donnan systems at equilibrium, for membranes containing numerous pores. In such membranes, the opposing pressure that acts from solution 2, as well as the attendant reduction of Δ*P* in solution 1 within the pores (Equation 4), reduces the contribution of the electric forces in the pores to the establishment of the pressure across membrane. On the other hand, the existence of a potential gradient on the side that contains the polymer leads to an increase in the concentration of the charged impermeant polymer at the interface with the membrane and thus increases the pressure that develops in this system. Clearly, the rules of thermodynamics dictate that the pressure that develops is the colloid osmotic pressure as long as the membranes are permeable to small ions but impermeable to the charged polymer. Nevertheless, the specifics of the physical mechanisms that operate in such membranes have yet to be elucidated.

## Additional Information

**How to cite this article**: Ilani, A. The development of pressure across membranes in Donnan systems. *Sci. Rep.* **5**, 14695; doi: 10.1038/srep14695 (2015).

## Supplementary Material

Supplementary Information

Supplementary dataset

## Figures and Tables

**Figure 1:**
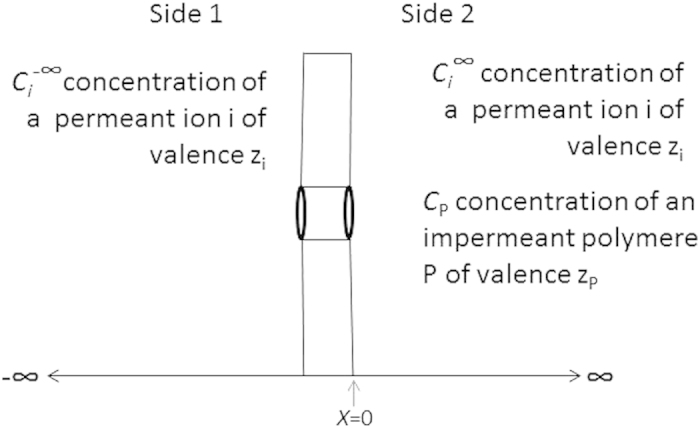
Scheme of a Donnan system. The value of *x* is set to 0 at the interface of a pore within the membrane with the solution on side 2. The charged polymer is excluded from the pore due to its size. The length of the pore, i.e., the thickness of the membrane, is >10^3^ Å. The membrane is permeable to solutes and water only due to the presence of its pores.

**Figure 2:**
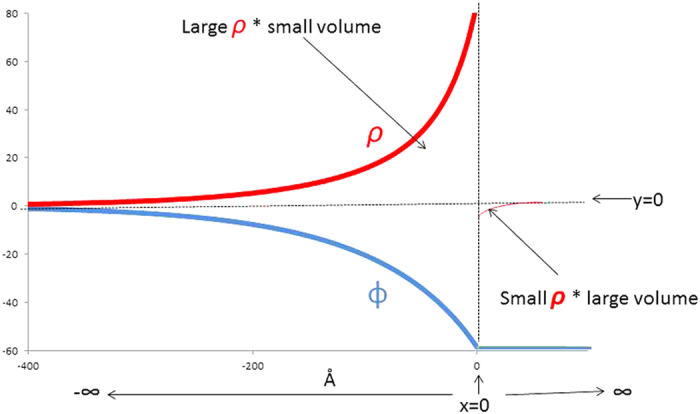
Course of electric potential, *ϕ*, and charge density, ρ, within a pore of a membrane in a Donnan system. The total cross-sectional area of the pores is very small compared to the area of the membrane, so that almost all the change in *ϕ* and ρ occurs within the pore itself. This diagram depicts a system containing a negatively charged impermeant polymer. Thus, if the potential at –∞ is set to 0, *ϕ* at +∞ is negative, while the *ρ* in the pore is positive. Electroneutrality requires that the total positive charge within the few pores to the left of the interface be equal to the total negative charge in the large volume to the right.
