# Supplementary material for: The development of pressure across membranes in Donnan systems
Source: Sci Rep. 2015 Oct 12;5:14695. doi: 10.1038/srep14695 (PMC4600974; doi:10.1038/srep14695)
Supplement: Supplementary Information [file srep14695-s1.pdf]

The Development of Pressure Across Membranes in Donnan Systems

Asher Ilani

Supplementary Information File 1

Numerical Analysis of Poisson Electric Equation for a Particular Donnan System

The Poisson electric equation was solved numerically for the bulk water-filled pores within the membrane. The system is homogenous with respect to the *y* and *z* coordinates, and so the Poisson equation takes the form:

$$d^2\phi/dx^2 = -\rho/(80\epsilon)$$

where ϕ is the electric potential, ρ is the charge density, ϵ is the permittivity of free space and *80* is the dielectric constant of water.

We present the numerical analysis for a particular Donnan system that comprises the following solutions:

On the left-hand side, i.e., at $x<0$,

$C_{-\infty}$(a monovalent counterion, e.g., C^{+})= 0.9 mM

$C_{-\infty}$(a monovalent coion, e.g., A^{-}) = 0.9 mM.

On the right-hand side, i.e. at $x>0$,

C_{∞}(a monovalent counterion, e.g. C^{+})= 8.1 mM

C_{∞}(a monovalent coion, e.g. A^{-}) = 0.1 mM

P_{∞}(charged polymer of valence z_{-P} = -8) = 1.0 mM.

The system is in equilibrium as evidenced by the equality of the products of the permeant cation and anion on the two sides of the membrane. As required, both solutions are electroneutral far from the membrane.

Equilibrium is maintained by a potential difference between the two sides of the membrane: if we set the reference potential $\phi(-\infty)$ to zero, the value of $\phi(\infty)$ is -58.7 mV at 37^{0}C. Since we assume that most of the equilibrium potential drop occurs across the pores within the membrane, the numerical solution of the Poisson equation is intended to describe the potential course within the pore from $x=-\infty$ to $x=0$. As the potential becomes more negative towards $x=0$, the concentration of the ions in that layer changes according to the Nernst equation:

$$C_i(\phi) = C_i(0)\ \exp(-z_i F\phi/RT) \qquad [2].$$

The procedure for the numerical solution shown here proceeded at sequential layers of water of thickness δx=1Å, after showing that further narrowing of δx does not affect the results of the analysis. We start at a layer remote from the membrane that is temporarily

set at x=0. At this layer, ϕ and $d\phi/dx$ are set to zero, whereas $d^2\phi/dx^2$ is set at a very small value. Generally, we applied the value of 10^{-5} mV/Å^2 to this initial layer. (Further reduction of the initial value of $d^2\phi/dx^2$ did not affect the course of the potential near the membrane; it just had the effect of moving the starting point farther away from the membrane). Then we move closer to the membrane by repetitive 1 Å layer steps. The *n*'th layer is, therefore, at a distance of *n* Å from the starting point. Using Excel, we determine for each layer of water the following parameters:

$\phi(n)$; This is determined as follows:

$$\phi(\mathrm{n}) = [d^2\phi/dx^2(n-1) + 2 * \phi(n-1) - \phi(n-2)] \qquad [3],$$

where $d^2\phi/dx^2$ is given in units of mV/Å^2

$[C^+](n)$ and $[A^-](n)$ are determined by the Nernst equation for 37^0C and are given in

10^{-3}M/L, which is equivalent to M/m^3.

$\rho(n)$ is equal to $\{[\mathrm{C}^+](n) – [A^-](n)\}$ in units of 96.5 C/L = 96500 C/m^3

$d^2\phi/dx^2\ (n) = \mathrm{k}\rho(n)$.

The proportionality constant k is the value of $d^2\phi/dx^2$ in mV/(Å)^2 when the ρ is 96500 C/m^3.

Calculation of k is shown below.

The procedure is stopped at n_{final} where $\phi(n_{\mathrm{final}})= \phi^{\mathrm{eq}}$ (or when absolute value of $\phi(n_{\mathrm{final+1}})$>the absolute value of ϕ^{eq}).

At this point it is possible to depict any of the calculated parameters as a function of their distance from the membrane, m, where the n'th layer is at a distance m ($m=n-n_{\mathrm{final}}$) Å from $x=0$, i.e., from the junction of pores and the solution on side 2. The curves for ϕ, ρ in Supplementary Figure S1 are taken from such an analysis for the particular Donnan cell described above. The curve for the pressure P in this Figure is based either on the numerical analysis or on the combined numerical analysis of ϕ as a function of x and the analytical results expressing the value of P as a function of ϕ (see text equation 5). In comparing the two methods we found that the maximal difference between the P values at a particular x were less than 2%.

The value of k was determined as follows:

$d^2\phi/dx^2 = -\rho/(80\epsilon)$

For $\rho = 96500$ C/m^3 $\quad d^2\phi/dx^2 = -96500/(80\epsilon)$

$\epsilon = 1/(36\pi * 10^9)$ in units of F/m; hence,

$d^2\phi/dx^2 = -96500/(80\epsilon) = 96500 * 36\pi * 10^9/80)$ in units of V/m^2.

To convert it to units of mV/Å^2 we have to multiply it by 10^{-17} since 10^3 mV = Volt, and 10^{20}Å^2=m^2.

Therefore, k = 0.001364 in units of mV/Å^2/mEq/L

The data of the numerical solution of the Donnan system described in Figure S1 is presented in the Supplementary Excel file.

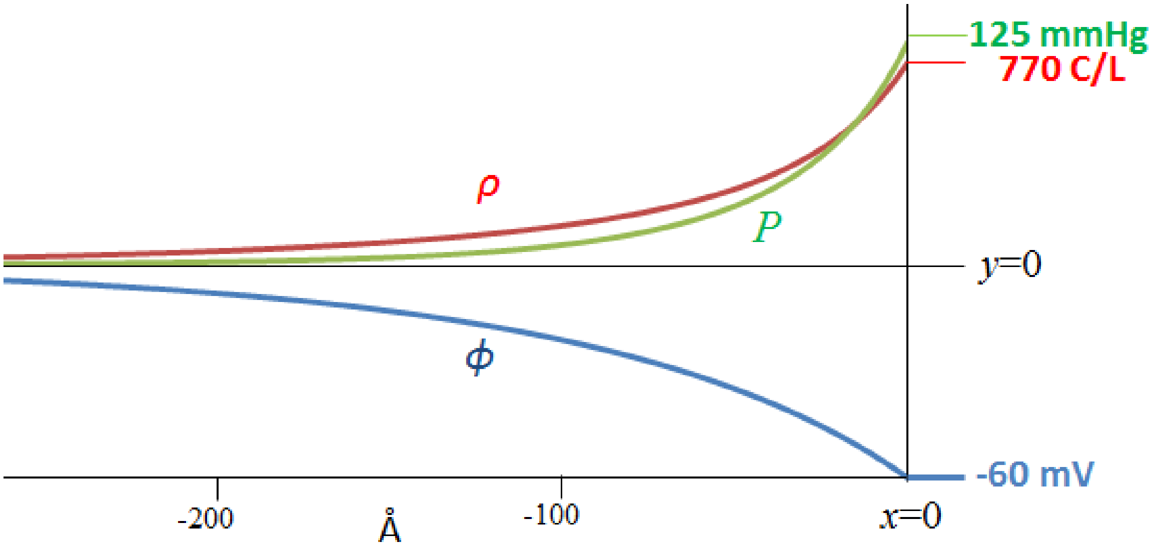

Supplementary Figure S1: The course of ϕ, ρ and P as a function of distance from the junction between the pores and the solution on side 2 (which is set at x=0) for the particular Donnan system described above. It is assumed that the system behaves ideally, i.e., activity coefficients of the solutes are close to unity. The value of P as a function of x was obtained by two ways: 1), by the numerical solution of the cumulative force acting on the charges in the pore and 2), from the analytical solution of $P(\phi)$ as expressed by equation 5 in the text and the numerical resolution of $\phi(x)$ as shown here; the difference between the two is below the resolution of the plot for all values of x<0.

Supplementary Information File 2

The Contribution of the Action of the Electric Forces within Pores to the Appearance of Pressure across Membranes in Donnan Systems

We begin by using the well-known expression for the Nernst potential, limiting ourselves to dilute solutions (where activity coefficients are close to unity), and consider the side containing only permeant ions (side 1 in text Figure 1):

$$C_i(\phi) = C_i(0)\ \exp(-z_i F\phi/\mathrm{RT}),$$

where C_i is the concentration of permeant ion i (mol/m^3), z_i is the valence, F is the Faraday constant (C/mol), R is the universal gas constant (J/K/mol), T is the absolute temperature (K), and ϕ is the potential (V); ϕ is set to 0 far from the membrane in the *negative* direction.

The charge density ρ (C/m^3) is

$$\rho = \textstyle\sum_\mathrm{i} C_i(0)\mathrm{z_i}F\ \exp(-\mathrm{z}_i F\phi/\mathrm{RT}),$$

so that the force δf (J/m or N) acting on a thin layer of solution of area A (m^2) and thickness δx (m) is

$$\delta f = -\ \rho\ (\mathrm{A}\delta x)\ \boldsymbol{(\delta\phi/\delta \mathbf{x})}$$

$$= -\textstyle\sum_\mathrm{i} C_i(0)\mathrm{z_i}F\ \exp(-z_i F\phi/\mathrm{RT})\ (\mathrm{A}\delta x)\ \boldsymbol{(\delta\phi/\delta \mathbf{x})}.$$

The pressure difference δP (N/m^2) across the layer is thus

$$\delta P = \delta f/A$$

$$= -\sum_i C_i(0) z_i F \exp(-z_i F\phi/RT)\, \delta\phi,$$

P being a function of *x* only through the dependence on ϕ, and

$$\delta P(\phi)/\delta\phi = -\sum_i C_i(0) z_i F \exp(-z_i F\phi/RT).$$

If now $\delta \rightarrow 0$, we can integrate $P(\phi)$ over ϕ to get

$$P(\phi) = RT \sum_i C_i(0) \exp(-z_i F\phi/RT) + \text{constant}.$$

The change in $P(\phi)$ when ϕ goes from 0 to its equilibrium value ϕ^{eq}, is thus simply

$$\Delta P = P(\phi^{eq}) - P(0) = RT[\sum C_i(\phi^{eq}) - [\sum C_i(0)].$$

Note that 1 mol/m^3, the units of C_i, is equivalent to 1000 mmol/1000 L or 1 mM.

The fact that ΔP is dependent on ϕ but independent of *x*, although ϕ is dependent on *x*, implies that the conclusion that ΔP accounts precisely for the difference between the osmotic values of the two solutions in a Donnan system is true even if there are minor discrepancies in the numerical values used for the various constants.
